# Habenula kisspeptin retrieves morphine impaired fear memory in zebrafish

**DOI:** 10.1038/s41598-020-76287-9

**Published:** 2020-11-11

**Authors:** Mageswary Sivalingam, Satoshi Ogawa, Ishwar S. Parhar

**Affiliations:** Brain Research Institute, Jeffrey Cheah School of Medicine and Health Sciences, Monash University Malaysia, Jalan Lagoon Selatan, Bandar Sunway, 47500 Subang Jaya, Selangor Malaysia

**Keywords:** Neuroscience, Learning and memory, Fear conditioning

## Abstract

The habenula is an evolutionarily conserved brain structure, which has recently been implicated in fear memory. In the zebrafish, kisspeptin **(**Kiss1) is predominantly expressed in the habenula, which has been implicated as a modulator of fear response. Hence, in the present study, we questioned whether Kiss1 has a role in fear memory and morphine-induced fear memory impairment using an odorant cue (alarm substances, AS)-induced fear avoidance paradigm in adult zebrafish, whereby the fear-conditioned memory can be assessed by a change of basal place preference (= avoidance) of fish due to AS-induced fear experience. Subsequently, to examine the possible role of Kiss1 neurons-serotonergic pathway, *kiss1* mRNA and serotonin levels were measured. AS exposure triggered fear episodes and fear-conditioned place avoidance. Morphine treatment followed by AS exposure, significantly impaired fear memory with increased time-spent in AS-paired compartment. However, fish administered with Kiss1 (10^–21^ mol/fish) after morphine treatment had significantly lower *kiss1* mRNA levels but retained fear memory. In addition, the total brain serotonin levels were significantly increased in AS- and Kiss1-treated groups as compared to control and morphine treated group. These results suggest that habenular Kiss1 might be involved in consolidation or retrieval of fear memory through the serotonin system.

## Introduction

Fear memory and fear responses are an essential part of an animal’s survival mechanism^1^. However, when fear responses are amplified and fail to extinguish and thus become debilitating even in the absence of threat, it can be classified as pathology in fear memory as in phobias and post-traumatic stress disorder^2^. There are accumulative evidences that acute morphine treatment has protective effect against fear-related disorders^3–5^. In rats, opioid receptor agonists including morphine administration blocks the development of stress-enhanced fear learning, acquisition of memory, and memory consolidation^6–9^. On the other hand, acute administration of opioid receptor antagonists facilitates the acquisition and prevents the extinction of fear conditioning^10–13^. In rodents, numerous studies have suggested that the amygdala is involved in the formation of fear memories^14, 15^. Microinjection of morphine into the amygdala impairs fear conditioning in rats^16^. In addition, the connectivity between the amygdala and meso-temporal structures including the hippocampus is also critical for the contextual elements of fear learning^2^. Chronic exposure to opiates causes learning deficits, which are dependent on the hippocampus^17, 18^. However, effect of morphine on the hippocampus-dependent cognitive processes remains controversial^19, 20^. Morphine acts through mu opioid receptor (MOR) to exhibit its effects^21^, but MOR is widely distributed throughout the brain and the action sites of morphine that are involved in fear conditioning are not fully defined. Secondary, morphine can influence a range of neurotransmitters implicated in fear conditioning^22–24^ and hence, morphine-induced fear memory impairment is expected to be modulated by multiple neural circuits and molecular signalling pathways^25, 26^.

In animal models, fear learning and memory can be studied by the Pavlovian fear conditioning, consisting of three phases: acquisition, consolidation, and reconsolidation (retrieval)^1^. The brain regions that are responsible for the fear memory and learning in teleost fish remains debatable, but successful induction of the Pavlovian fear conditioning in teleost fish species indicates the presence of neuronal mechanism or pathway that processes the development of fear conditioning in fish brain^27–29^. In goldfish, lesions in the medial telencephalic pallium (MP) impair active avoidance learning^30^. Further, a recent study in zebrafish has identified a subpopulation of neurons in the medial zone of the dorsal telencephalon, a part of the MP, essential for fear conditioning^29^. On the other hand, classical aversive conditioning remains spared even after ablation of the entire telencephalon in goldfish^31^. Therefore, in addition to the telencephalic region, other brain regions are also involved in fear learning and memory in fish^32^.

Recent studies in mammals have implicated the habenula-interpeduncular nucleus (IPN) as one of the pathways that is involved in fear memory^33, 34^. The habenula is an evolutionarily conserved epithalamic structure that is involved in motivation and emotional decision making^35^. In mammals, the habenula consists of the medial (MHb) and lateral (LHb) subnuclei, and the MHb has been implicated in fear extinction^33, 36^, while the LHb in temporal stability of contextual fear and spatial memory^34, 37^. Although the role of habenula in opioid-induced fear memory impairment has not been demonstrated, the habenula has been implicated as one of the opioid-sensitive brain regions because of high expression of MOR^38–41^. We have recently shown dense expression of MOR in the habenula of zebrafish (*Danio rerio*), and exposure to morphine suppresses neural activity in the habenula^42^. We have previously shown the expression of a reproductive neuropeptide, kisspeptin (Kiss1) and its cognate receptor (GPR54 = Kiss1R) in the ventral habenula (vHb, homologous to mammalian LHb) in the zebrafish^43, 44^. In addition, habenular Kiss1-Kiss1R signalling has been shown to modulates odorant cue (alarm substances = AS)-induced fear responses via serotonergic signalling^45^. A more recent study in larval zebrafish has shown that genetic ablation of *kiss1* impairs avoidance learning^46^, affirming the role of habenula Kiss1 in fear learning or memory. However, whether the habenular Kiss1 neurons are involved in the morphine-induced fear memory impairment remains unclear.

Therefore, in the present study, we first validated whether morphine impairs AS-induced fear conditioning using the conditioned place avoidance paradigm^47^. Next, to elucidate the role of habenular Kiss1 signalling in fear conditioning, effect of centrally administered Kiss1 on fear memory consolidation and morphine-induced fear memory impairment were examined. Finally, to assess the possible involvement of serotonin (5-HT) in morphine-induced fear memory impairment, the total content of 5-HT in the brain was quantified using LC–MS/MS.

## Results

### Effect of morphine on fear memory

**Figure 1:**
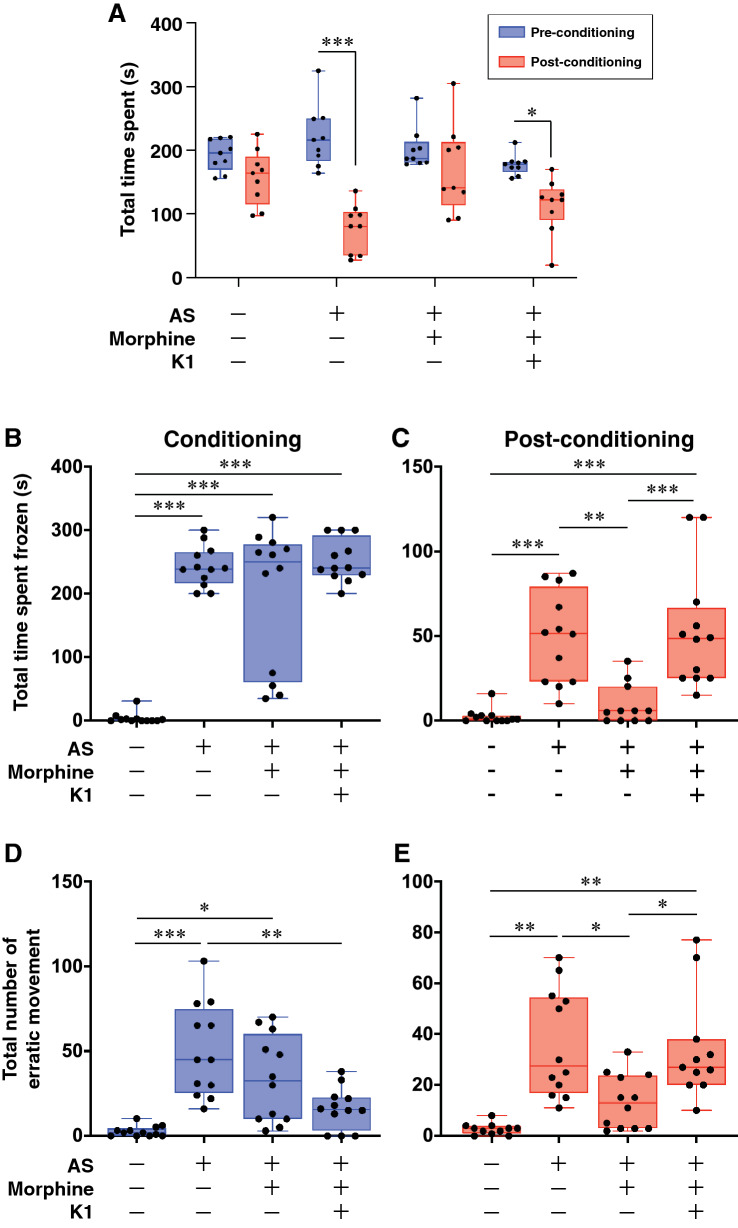
Effects of morphine on AS-induced fear conditioning and Kiss1-induced fear memory retrieval. (**A**) Graph showing the change in preference between pre- (*red* columns) and post-conditioning (*blue*-columns) in different treatment groups (Groups 1–4, n = 12 per group), which were assessed based on their total time spent in AS-conditioned (originally preferred) compartment as compared to the initial preference. Data are presented as mean ± SEM. Independent t-test comparisons between pre- and post-conditioning phase. (**B**,**C**) Graph showing freezing duration (seconds) in different treatment groups during the conditioning (**B**) and post-conditioning (**C**). (**D**,**E**) Graph showing total numbers of erratic movement during the conditioning (**D**) and post-conditioning (**E**). Data are presented as mean ± SEM and analysed using one way ANOVA followed by Tukey’s multiple comparison test. *P < 0.05; **P < 0.01, ***P < 0.001.

To validate the effect of morphine on fear conditioning, fish were exposed with morphine and examined AS-induced fear conditioning using the conditioned place avoidance paradigm. Fish that were exposed to a single dose of conspecific AS (2 ml of AS solution per tank) during the conditioning phase successfully exhibited clear AS-induced fear response including increase in freezing [F_(3, 44)_ = 44.80, p < 0.00001, gεs = 0.75, 95% CI (0.58, 0.84), Fig. 1B] and erratic behaviour [F_(3, 44)_ = 13.22, p < 0.00001, gεs = 0.47, 95% CI (0.22, 0.64), Fig. 1D] as compared to Non-AS-treated control group. After the fear conditioning, the fish showed significant (p = 0.000003, Cohen’s d = 3.293747, Fig. 1A) reduction in their time spent in the compartment paired with AS during the post-conditioning phase, as compared to Non-AS (control) fish, indicating a switch from their preferred compartment due to AS-induced fear memory. They also exhibited fear behaviours including freezing [F_(3, 43)_ = 14.97, p < 0.0001, gεs = 0.51, 95% CI (0.25, 0.67), Fig. 1C] and erratic movements [F_(3, 44)_ = 9.473, p < 0.0001, gεs = 0.39, 95% CI (0.14, 0.57), Fig. 1E] when they entered the AS-paired compartment. Thus, AS-treated fish successfully elicited AS-conditioned avoidance behaviour. In contrast, morphine-treated fish showed no difference in the time spent in the compartment paired with AS during pre- and post-conditioning [p = 0.219, effect Cohen’s d = 0.602041), Fig. 1A]. Also, the freezing time was significantly reduced as compared to AS-exposed fish during the post-conditioning phase (p = 0.001, Fig. 1C). However, there was no significant difference in the number of erratic behaviour phases (p = 0.0447, Fig. 1E) as compared to AS-treated fish during post-conditioning. Overall morphine-treated fish diminished AS-induced freezing response and AS-conditioned fear avoidance.

### Effect of Kiss1 administration on morphine-induced fear impairment

To examine the moderating effect of Kiss1 on morphine-induced fear memory impairment, Kiss1 peptide (kisspeptin1-15, 10^–21^ mol/fish), the dose that has been shown to depolarize vHb neurons^46^, was centrally administered to morphine-treated fish after AS-conditioning. Kiss1-treated fish showed active avoidance to the AS-paired compartment (p = 0.00076, Cohen’s d = 1.953633, Fig. 1A). In addition, total freezing time [F_(3, 43)_ = 14.97, p = 0.0003, gεs = 0.51, 95% CI (0.25, 0.67), Fig. 1C] and total number of erratic movements [F_(3, 44)_ = 9.473, p = 0.0078, gεs = 0.39, 95% CI (0.14, 0.57), Fig. 1E) were significantly increased in Kiss1 treated fish as compared to the morphine-treated group during the post-conditioning phase. This result suggests that Kiss1 peptide treatment diminished the inhibitory effect of morphine on fear memory consolidation or memory retrieval.

### *kiss1* mRNA expression in the brain

**Figure 2:**
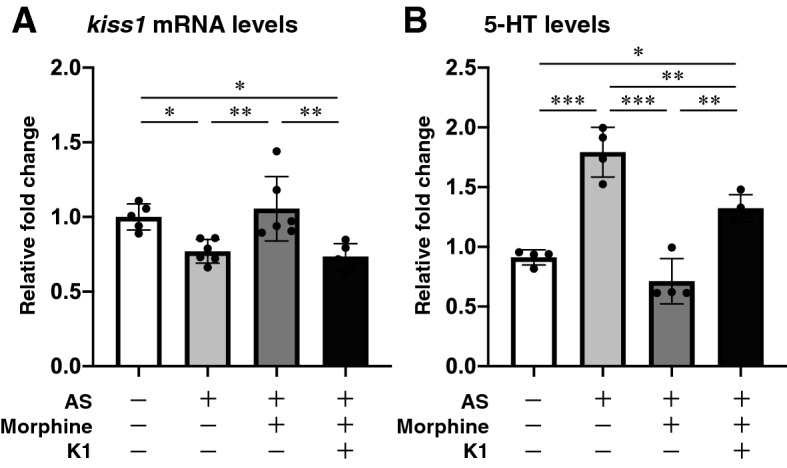
Effect of morphine and Kiss1 administration on *kiss1* mRNA and 5-HT levels in the brain. Graph showing the *kiss1* mRNA (**A**) and 5-HT levels (**B**) in different treated groups (n = 4–5 per group). Data are presented as mean ± SEM and analysed using one way ANOVA followed by Tukey’s multiple comparison test. *P < 0.05; **P < 0.01, ***P < 0.001.

To elucidate the effect of AS, morphine and exogenous Kiss1 on habenula Kiss1 neural activity, *kiss1* mRNA levels were quantified [F_(3, 18)_ = 7.958, gεs = 0.57, 95% CI (0.15, 0.76)]. In the brain of AS + vehicle-treated group, *kiss1* mRNA levels were significantly lower as compared to those in Non-AS (controls) group (p = 0.0494) and AS + morphine-treated group (p = 0.0084, Fig. 2A). Similarly, in the brain of AS + morphine + Kiss1-treated group, *kiss1* mRNA levels were significantly lower as compared to those in Non-AS (controls) group (p = 0.0279) and AS + morphine-treated group (p = 0.0049, Fig. 2A). However, there was no difference in *kiss1* mRNA levels between Non-AS controls and AS + morphine-treated group (p = 0.9034, Fig. 2A). Since habenula Kiss1 neurons are negatively regulated through an autocrine mechanism^44^, the low levels of endogenous *kiss1* mRNA may indicate activation of endogenous Kiss1 peptide secretion in the habenula. Further, as morphine suppresses neural activity in the habenula^42^, morphine exposure could diminish the fear memory consolidation process via inhibition of vHb neural activities or disruption of Kiss1 secretion.

### Effect of morphine and Kiss1 administration on 5-HT levels in the brain

To examine the role of 5-HT in fear responses and fear conditioning, the total brain 5-HT levels were quantified under various treatments [F_(3, 12)_ = 38.21, gεs = 0.91, 95% CI (0.67, 0.96)]. In the brain of fish exposed to AS, total 5-HT levels were significantly higher than Non-AS (controls) group (p < 0.0001, Fig. 2B]. In contrast, fish treated with morphine followed by AS-exposure, 5-HT levels were significantly lower as compared with AS-exposed fish (p < 0.0001, Fig. 2B). However, in fish administered with AS and morphine followed by Kiss1 administration, the 5-HT levels were significantly higher as compared to AS + morphine-treated group (p = 0.0006), although they were still significantly lower than those in fish treated with AS alone (p = 0.0051, Fig. 2B). These results indicate that morphine-induced fear memory impairment could be due to the lower levels of 5-HT. Further, this suppression of 5-HT levels could be modulated by lower levels of Kiss1 or inhibition of vHb neural activities.

## Discussion

In this study, alarm substance (AS) successfully stimulated fear response. While fish treated with morphine exhibited a significant reduction of fear response in the AS-paired compartment and its associated conditioned avoidance, indicating that morphine treatment diminished AS-conditioned fear learning and memory. On the other hand, fish administered with Kiss1 peptide following morphine treatment displayed a significant increase in conditioned avoidance and fear responses in the AS-paired compartment. These results suggest that Kiss1 treatment successfully retrieved AS-conditioned fear memory that was impaired by morphine.

Following AS exposure in the preferred compartment, the freezing duration and the total number of erratic movements was significantly increased, confirming successful induction of fear response by AS exposure. In addition, because AS-exposure increases fear responses to conditioned stimulus (CS) after paired with a neutral visual stimulus (colour), AS can be considered as an unconditioned stimulus (US) according to the Pavlovian fear conditioning paradigm^47, 48^. After the conditioning to AS-induced fear, the total time spent in the non-preferred compartment was significantly increased. Since animals changing their preference to the “safer” compartment can be defined as signs of avoidance learning^47^, the significant reduction of time spent in the AS-paired compartment indicates successful avoidance conditioning to AS. In the present study, behavioural parameters observed were not distinguished between the preferred and non-preferred compartment. However, the fish spent significant time in the non-preferred compartment due to conditioned avoidance, hence, the behavioral parameters quantified are likely to have originated from the non-preferred chamber. In rodents, the protective effect of morphine on fear memory and responses appears after 6 h but not immediately after the trauma given, and the most effective response is observed when morphine treatment is given 24 h after the traumatic event^49^. However, in the present study, single morphine treatment 30-min after AS-induced fear stimuli successfully reduced expression of AS-conditioned avoidance of fish, which is much shorter time than that in rats. Although the critical time scale that is needed for successful fear conditioning in adult zebrafish remains unknown, the dose and timing of morphine treatment was sufficient to suppress the fear learning.

Under the Pavlovian fear conditioning paradigm, fear conditioning process consists of several phases: the acquisition, consolidation, temporal stability and the reconsolidation (retrieval) of fear memory, which are modulated by distinct molecular processes^26^. Clinical and preclinical studies have hypothesized that morphine administration following a trauma interrupts memory consolidation^5, 8, 50, 51^. In addition, microinjection of morphine into the amygdala that is responsible for convergence of conditioned stimulus and unconditioned stimulus, also results in fear memory impairment^16^. Our preliminary experiment with a single dose injection of morphine an hour after the fear conditioning demonstrated lesser inhibition of fear responses (data not shown). Hence, we applied repeated morphine treatments before (Day-2) and after (Day-3) the consolidation. However, morphine has also been implicated in impairment of fear memory persistence and retrieval^52^. Therefore, it is also possible that the successful induction of fear memory impairment by morphine exposure could be due to its effect on memory retrieval, which remains to be further validated.

Our results show that administration of Kiss1 significantly reduced *kiss1* mRNA levels indicting autocrine negative feedback regulation of habenula Kiss1 neurons as demonstrated previously^44^. In addition, in fish exposed AS, *kiss1* mRNA levels were reduced, while morphine treatment diminished this reduction. Although association between endogenous levels of *kiss1* mRNA and Kiss1 peptide is not clear, low *kiss1* mRNA levels may imply the higher levels of Kiss1 peptide or activation of peptide secretion. On the other hand, a recent electrophysiological assay in larval zebrafish has shown that Kiss1 exhibits concentration-dependent dual (stimulatory and inhibitory) effects on vHb neural activity, whereby vHb neurons are depolarized at low concentrations (10 nM and 100 nM), whereas they are hyperpolarized at high concentrations (1 µM and 5 µM)^46^. In fact, *c-fos* expression in the vHb was only induced by a concentration of 10^−11^ mol/fish of Kiss1 peptides, but not with a higher concentration of 10^−9^ mol/fish^44^. Hence, the dose of Kiss1 utilized in the present study (10^–21^ mol/fish in 1 µl), is expected to depolarize vHb neurons, which could be associated with reduction of *kiss1* mRNA levels or Kiss1 peptide secretion activity.

In mammals, habenula activation itself has shown to induce conditioned avoidance^53^. In another study in rodent, inactivation of LHb before inducing aversive learning impairs the temporal stability of fear memory, but it does not block memory consolidation^54^. A recent study in zebrafish has shown that optogenetic stimulation of vHb neurons alone can evoke conditioned place avoidance^55^. Hence, the impaired AS-conditioned fear memory by morphine could be successfully retrieved by activation of vHb neurons by Kiss1 administration. However, it is also possible that Kiss1 administration could have blocked the morphine-induced signalling within the habenula, which resulted in the retainment of the fear memory. Further, it is also possible that Kiss1 administration may influence on decision-making process as the habenula is also known to be involved in transforming sensory signals to negative emotion and behavioural avoidance^56^. In rats, anxiety-like state closely associates with decision-making process^57^. Our previous study has demonstrated that administration of Kiss1 peptides increases the top–bottom (up-down) transition behaviour^45^, which is known as a behavioural parameter to assess anxiety in zebrafish^58^. However, other than this behaviour, we did not observe any effect of Kiss1 on anti-anxiety-related behaviours^45^. Therefore, the change in preferred compartment by Kiss1 administration may not be due to its potential anxiolytic effect on emotional state of fish.

In the present study, we examined the effect of Kiss1 administration on morphine-induced fear impairment 24 h after administration. However, it remains unknown when Kiss1 actually attenuated the effect of morphine, and how long the effect of Kiss1 administration on fear memory consolidation can last. We have previously shown that induction of swimming hyperactivity of fish by Kiss1 administration occurs at 4 h but not at 1 h post administration^45^, indicating a cascade of molecular events may take place before presenting the effect of Kiss1 on behavioural output. In rodents, treatment with kisspeptin-13 (a derivative of prepro-kisspeptin containing 13-amino acids) affects passive avoidance behaviour 24 h after administration indicating that the effect can last over a day^59^. In rodents and zebrafish, habenula activation can induce conditioned avoidance^53, 55^, however, the lasting effect of habenula activation has not been demonstrated. Although the precise mechanism underlying the re-consolidation of morphine-impaired fear memory by Kiss1 administration remains unclear, habenular Kiss1 seems to act as a mediator in retrieval of the consolidated memory.

We observed elevated brain 5-HT levels by AS-exposure as reported previously^60^. In monkeys, serotonergic raphe neurons encode reward expectation values, which may also be utilized for fear learning and avoidance^61–63^. Previous studies have shown the role of vHb-MR pathway in the regulation of serotonergic neurons in response to avoidance learning in zebrafish^46, 55^. Further, habenular Kiss1-KissR1 pathway has been shown to positively modulate 5-HT levels in the zebrafish^44^. Moreover, AS-induced freezing and erratic behaviours are modulated by serotonergic signalling^64^. Interestingly, in *kiss1* gene mutant zebrafish, activation of serotonergic raphe neurons by an electric stimulus is impaired^46^, implicating the possible role of Kiss1-serotonin signalling in avoidance learning. In the present study, the elevation of 5-HT in AS-conditioned fish was attenuated by morphine treatment. Hence, it can be hypothesized that suppression of 5-HT levels in morphine-treated fish could be modulated via inhibition of habenular Kiss1 neurons, which results in fear memory impairment.

## Conclusion

In the present study, morphine treatment impaired fear memory by decreasing avoidance to the AS-paired compartment. On the other hand, administration of low concentration of Kiss1 peptide following morphine treatment disinhibited the morphine-induced fear memory impairment. 5-HT levels in the brain were increased upon AS exposure, but decreased by morphine treatment. Thus, it can be speculated that the vHb (Kiss1)-MR (serotonin) pathways might be involved in consolidation or retrieval of fear memory.

## Methods and materials

### Experimental animals

Adult male (3–6 months old), in-house bred wild-type AB strain zebrafish (obtained from Institute of Molecular and Cell Biology, Singapore) were maintained in groups of 10 fish per 20 L freshwater aquaria (home tank) at 28 ± 0.5 °C with a controlled natural photo regimen (14/10 h, light/dark) as described previously^42^. Adult zebrafish diet (purchased from Zeigler, Gardners, PA, USA) were fed twice daily.

### Ethical statement

This study was carried out in strict accordance with the recommendations in the Guidelines to promote the wellbeing of animals used for scientific purposes: The assessment and alleviation of pain and distress in research animals (2008) by the National Health and Medical Research Council of Australia (https://www.nhmrc.gov.au/guidelines-publications/ea18). All the experiments in this study were conducted following the ethical approval of Monash University Animal Ethics Committee (Project approval number: MARP/2017/049).

### Alarm substance (AS) extraction

Conspecific alarm substance (AS) was extracted from zebrafish as described previously^45^. Briefly, fish were rapidly euthanized by submerging them in ice-cold water and 10–15 superficial, shallow cuts were made on each side of the trunk of fish with a razor blade, and the cuts were immersed into 10 ml of distilled water for 1-min and collected it as an AS solution (10 ml per fish).

### AS-induced fear conditioning

**Figure 3:**
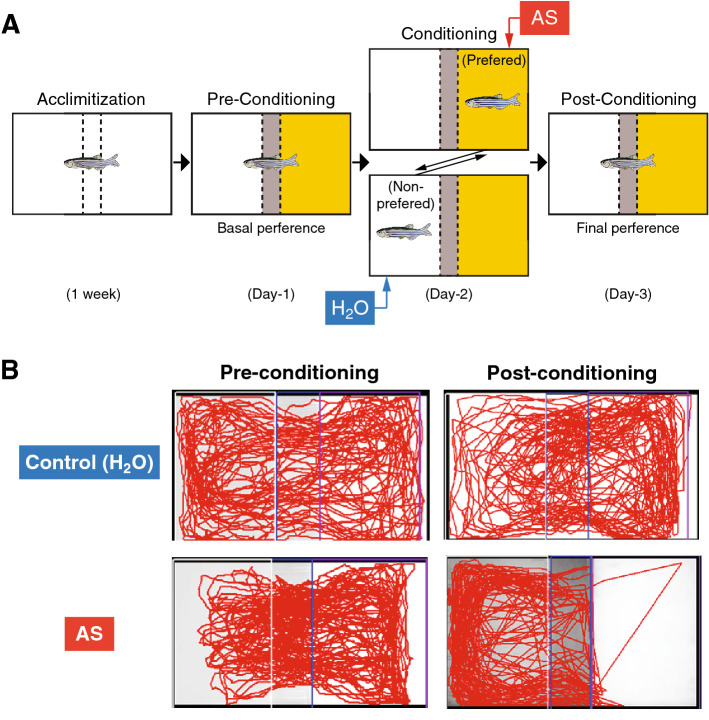
Alarm substance-induced fear conditioning. (**A**) Schematic drawing of conspecific alarm substance (AS)-induced fear conditioning paradigm. During pre-conditioning (Day-1), fish was given a choice for their preferred colour, either yellow or white coloured compartment (basal preference). After conditioning to AS-induced fear responses (Day-2), their change in preference was assessed based on their total time spent in AS-paired (originally preferred) compartment as compared to the initial preference (Day-3). (**B**) Representative top-view video tracking of swimming behaviour comparing between pre-conditioned (left) and post-conditioned (right) of control (Non-AS, water-treated, upper panels) and AS-treated fish (lower panels).

AS-induced fear conditioning was assessed by the conditioned place avoidance paradigm according to the procedure established by Maximino and co-workers^47^, which consists of 3 phases; pre-condition on day 1 after a week of acclimatizing to assess basal preference, conditioning phase on day 2 with AS-induced aversive experience, and post-conditioning phase on day 3 to determine the final preference (Fig. 3A).

#### Behavioural apparatus

Briefly, a tank [31 cm length (L) × 16 cm width  (W) × 20 cm height  (H)] divided into three-compartments using a lightweight board made of corrugated plastic with yellow and white [13 cm (L) × 16 cm(W) × 20 cm(H)], and grey [5 cm(L) × 5 cm (W) × 20 cm(H)] as chosen colours by a grey central divider (Fig. 3A). Top view of fish swimming behaviour was recorded by a video camera (Sony Handycam DCR-SX83E) that was positioned approximately 1 m above the tank. The behavioural experiments were conducted between 1100 to 1600 h, under the similar water temperature (28 ± 0.5 °C) and lighting (802.4 lx illumination) conditions to the home tank. Recorded video data were analysed using a behaviour tracking software, LoliTrack 2.0 (Loligo Systems, Tjele, Denmark).

#### Acclimatization

A week prior to behavioural study, fish were randomly taken from the housing tank and transferred to acclimatizing tank that was divided into 2 equal compartments by a transparent divider to allow visual contact by conspecifics to minimize isolation stress. To reduce handling stress, fish were netted and transferred to a beaker during changing once daily for about less than 5 min throughout the acclimatization period. The condition of the water, temperature and light were maintained as in the housing tanks.

#### Pre-conditioning phase (Day-1)

One day before the fear conditioning, the fish was individually placed into the central compartment (grey) of the apparatus. After 30 s of acclimatization period, the separators which block the yellow and white compartment were removed to allow fish move freely for 5 min followed by 6 min of video recording to assess the basal preference by measuring the time spent in each compartment. The compartment in which the fish spent more than 3-min was considered the preferred compartment and the other side of the compartment was considered as non-preferred compartment.

#### Conditioning (Day-2)

For fear conditioning, the fish was individually placed into its predetermined preferred compartment with the separators, and after 5 min of familiarisation period, 2 ml of AS solution was delivered in tank water via pipet, followed by 5-min of video recording. After the AS exposure, the fish was immediately transferred into a holding tank and the experimental tank was washed to remove the AS residue. The fish was then transferred to a non-preferred compartment of the experimental tank. After 5 min settling time, 2 ml of water (control) was delivered followed by 5-min of video recording and returned to their respective housing (acclimatisation) tanks.

#### Post-conditioning (Day-3)

To assess if the fear conditioning was successfully established, on the 3rd day of testing, the avoidance to the fear-conditioned compartment was assessed in the absence of AS. The fish was placed in the centre compartment with the separators. After 30 s of familiarisation period, the separators were removed to allow fish move freely for 5 min followed by 6 min of video recording to assess the basal preference by measuring the time spent in each compartment.

#### Behavioural parameters (Days-2 and -3)

During the conditioning period, AS-induced fear-related parameters such as number of erratic movement (sharp changes in the direction or velocity of swimming and repeated rapid darting), total freezing time (complete cessation of movement for 1 s or longer)^65^, and total distance swam were assessed. During the post-conditioning periods, in addition to those fear-related parameters, avoidance to the AS-paired compartment was assessed by comparing the time spent in the pre-conditioning and the post-conditioning phase for 6 min. After the completion of post-conditioning, the fish were killed by immersing them in water containing benzocaine (0.1 g/200 ml water; Sigma) and the whole brain samples were collected for measurement of *kiss1* gene expression and serotonin content levels.

### Treatment groups

**Figure 4:**
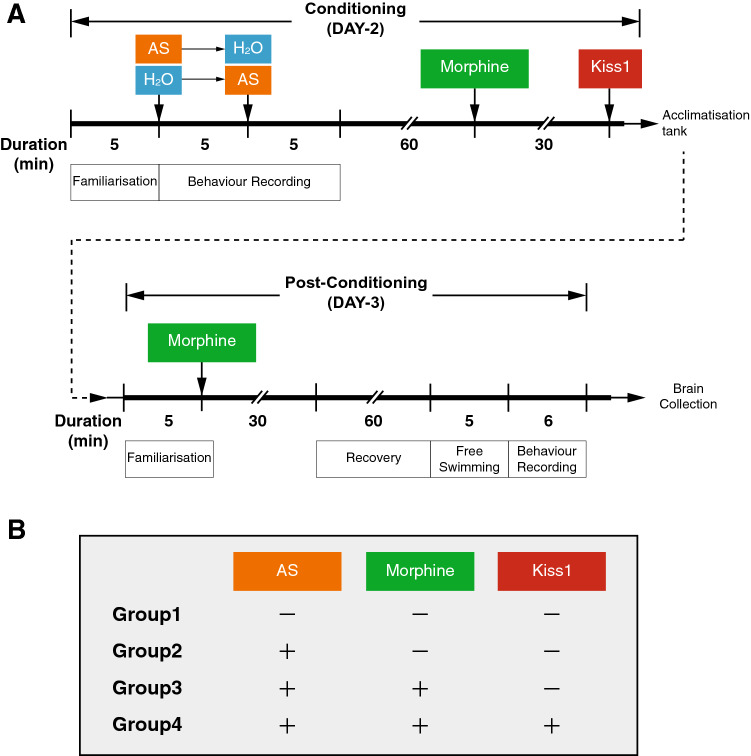
Treatment timeline and treatment groups during the fear conditioning. (**A**) Schematic of treatment timeline during the fear conditioning. On Day-2, the fish was transferred to either the preferred compartment or non-preferred compartment (at random), and control fish were treated with 5-min of water in preferred and non-preferred compartments (Non-AS, Group 1). Treatment groups (Groups 2–4) were exposed to alarm substance (AS) or water, respectively for 5-min. After 5-min treatment, the fish was transferred to the other side of compartment (preferred to non-preferred or vice versa) and exposed with AS or water for 5-min followed by transferring to the acclimatisation tank (AS-treated group, Group 2). For morphine-treated group (Groups 3 and 4), after one hour of recovery from fear conditioning, the fish were then treated with morphine (2 mg/l) or vehicle (water) for 30-min and transferring to the acclimatisation (housing) tank. On Day-3, the fish were placed in the centre compartment of experimental tank with the separators. After familiarisation period, the fish was again treated with morphine before the post-conditioning test. For Kiss1-treated group (Group 4), morphine-treated fish were centrally administered with 1 µl of Kiss1 (zebrafish kisspeptin1-15) at the dose of 10^–21^ mol/fish or 1 µl of water (control) before transferred to the acclimatisation (housing) tank. Four different treatment groups were summarized in (**B**).

To examine the effect of morphine and Kiss1 on AS-induced fear conditioning, the following four treatment groups (n = 10–12/group, Fig. 4A,B) were set: Group1, control (Non-AS): fish were only treated with MQ water throughout the experiments; Group2, AS + vehicle: fish treated with AS or water (control) in random order during conditioning; Group3, AS + morphine: fish treated with AS during conditioning followed by morphine exposure; and Group4, AS + morphine + Kiss1: fish treated with AS during conditioning followed by morphine treatment and then intracranial administration with Kiss1 peptide solution before the fish were returned to acclimatisation tank.

#### Morphine treatment

To examine the effect of morphine on fear conditioning, fish were exposed with morphine (Groups 3 and 4, Fig. 4B) during the conditioning and post-conditioning phase (Fig. 4A). Briefly, on the Day-2, after 5-min of conditioning with AS and 60-min of recovery (consolidation), fish was immersed in a 1 L container containing 800 ml of morphine (2 mg/l, morphine sulfate pentahydrate, Lipomed AG, Switzerland) for 30 min and transferred to the housing tank or proceed for Kiss1 administration. Next day (Day-3), prior to post-conditioning assessment, the fish was again immersed in morphine solution for 30 min. This was because the protective effect of morphine on fear memory and responses has been demonstrated to be most effective 24 h or 48 h after the traumatic event^6, 49^. The dose for morphine was chosen based on a previous study in adult zebrafish^66^.

#### Intracranial administration of Kiss1

To examine the effect of Kiss1 on morphine-induced fear memory impairment, fish (Group 4, Fig. 4B) were administered with Kiss1. Intracranial administration of Kiss1 was carried out as described previously^44^. Briefly, fish were anesthetized by immersion in 0.01% benzocaine (Sigma, USA) solution and placed on a sponge soaked with water. The cranial bone at the telencephalon-diencephalon border, close to the habenula above the left side of the anterior part of the optic tectum was incised using a sterilized barbed-end needle (30G × 1″ Terumo). Then, through this incision, the fish was intracranially injected with 1 µl of zebrafish kisspeptin1-15 (pyroglut-NVAYYNLNSFGLRY-NH2; Open Biosystems) at the dose of 10^–21^ mol/fish or MQ water (control) by heat-pulled glass capillary micropipette (inner diameter: 1 mm, model G-1, Narishige, Japan) attached with microinjector (IM-9B; Narishige). The dose of Kiss1 utilized in the present study (10^–21^ mol/fish in 1 µl) was chosen based on previous study^46^, which has been demonstrated to depolarize ventral habenula neurons in zebrafish.

### Gene expression assay

Gene expression level of *kiss1*mRNA in all the four groups were examined by real-time PCR as described previously^44^. Threshold cycle value (Ct) of *kiss1* gene was determined and then normalised to the mRNA levels of the housekeeping gene (*β-actin*). The data were then analysed according to 2^−ΔΔCt^ relative gene expression quantification.

### Quantification of brain serotonin levels by LC–MS/MS

Serotonin (5-hydroxytryptamine, 5-HT) levels in whole brain samples were determined using liquid chromatogram (LC)-double mass spectrometry (MS/MS) (LC–MS/MS) following the procedures previously developed by^67^.

#### Reagents

All solvents were of high-performance LC grade. Acetonitrile, formic acid and serotonin were obtained from Sigma Aldrich (St. Louis, MO, USA). Reagents for the analytical solution were prepared using 50% Acetonitrile with 0.1% formic acid, which was supplemented with 0.111 M ascorbic acid to prevent oxidation of analytes. The 5-HT standard solutions (1.25, 2.5, 5, 10, 20, 40, 80, 160 ng/ml) were prepared in the analytical solution containing 20 pg/µl of isoproterenol (Nacalai Tesque, Japan) as an internal standard.

#### Sample preparation

The dissected whole brain was homogenized in 100 µl of the analytical solution supplemented with 20 pg/µl of isoproterenol using micropestle for 60 s on ice. The homogenate was centrifuged at 1300G at 4 °C for 15 min and the supernatant was transferred to a filtration column (Cosmospin Filter G; hydrophilic PTFE membrane filter with 0.2 μm pores, Nacalai Tesque) and then centrifuged at 3000G at 4 °C for 30 min. Five microliters of the filtered supernatant was subjected for the LC–MS/MS analysis.

#### LC–MS/MS analysis

LC–MS/MS analysis was performed using Agilent Technologies 6410 Triple Quad LC/MS equipped with a Zorbax SB-C18 column (Narrow-Bore, 2.1 × 150 mm, 3.5 μm column; Agilent Technologies) as described previously^67^. Data acquisition and calculations were performed with Agilent Masshunter Quantitative Analysis software (RRID: SCR_015040, Agilent Technologies).

### Statistical analysis

IBM statistics V22.0 (IBM SPSS Statistics for Windows, Version 22.0. Armonk, NY: IBM Corp) was used to perform the statistical analysis. Total time spent in either preferred or non-preferred compartment at pre- and post-conditioning sessions were compared by unpaired Student’s t test and effect sizes were reported as Cohen’s d. Behavioural data (freezing time and number of erratic movements) and *kiss1* mRNA/5-HT levels analysed among different treatments were compared using one-way ANOVA followed by Tukey’s multiple comparison test with a single pooled variance and effect sizes were reported as generalized eta squared (gεs) with 95% confidence interval (CI) level. Data were expressed as mean ± SEM with the significance set at P < 0.05.
